# DOA Estimation for Underwater Target by Active Detection on Virtual Time Reversal Using a Uniform Linear Array

**DOI:** 10.3390/s18082458

**Published:** 2018-07-29

**Authors:** Haixia Jing, Haiyan Wang, Zhengguo Liu, Xiaohong Shen

**Affiliations:** 1 School of Marine Science and Technology, Northwestern Polytechnical University, Xi’an 710072, China; haixjing@gmail.com (H.J.); xhshen@nwpu.edu.cn (X.S.); 2 School of Engineering, Xi’an International University, Xi’an 710077, China; 3 China Shipbuilding Industry Corporation, Beijing 100097, China; liuzg@csic.com.cn

**Keywords:** active detection on virtual time reversal (ADVTR), direction of arrival (DOA) estimation, multipath effects, ULA, Capon algorithm, spatial smoothing

## Abstract

Aiming at addressing the problem caused by multipath effects in direction of arrival (DOA) estimation for underwater targets, a method based on the active detection on virtual time reversal (ADVTR) Capon algorithm is proposed. Unlike the conventional passive target estimation method ignoring the multipath effects but only considering the direct wave, the proposed method is closer to the actual situation in that the multipath signal propagation model is fully taken into account; in addition, active detection (AD) and virtual time reversal (VTR) processes are added, which use active detection to estimate channels, and virtual time reversal to realize focusing in a computer after the source-receive array (SRA) receives the reflected signal of the target. The combination of the two methods can greatly improve the energy of SRA and the precision of target direction estimation. With the popular acoustic field simulation tool Bellhop, the model proposed in this paper is verified. Compared with the conventional Capon method without time reversal, the simulation results show that the ADVTR Capon estimation method is far better, in terms of resolution and suppressing the sidelobes. It is suitable for the target DOA estimation under low signal-to-noise ratio (SNR) conditions. Further, we also show the ADVTR Capon estimation method works well in a real tank experiment.

## 1. Introduction

Direction of arrival (DOA) estimation is an important part of the target parameter estimation which has found broad applications in radar, sonar, wireless communication and so on [1]. There have been a large number of DOA estimation algorithms in the past few decades, including Conventional Beamforming, Capon Minimum Variance Method (MVM), Multiple Signal Classification (MUSIC, Estimation of Signal Parameters via Rotational Invariance Techniques (ESPRIT, Maximum Likelihood (ML) estimation, Weighted Subspace Fitting (WSF) algorithm, etc. [2]. Compressed Sensing (CS) [3,4] DOA estimation technology proposed in recent years also shows good performance with little less snapshots [5,6]. In addition, there are various DOA estimation methods for different applications and environments, such as those described in [2,7,8].

The aforementioned methods are usually based on the traditional direct-path-only assumption, where multipath is ignored or considered detrimental and a negative whose effect impacts the performance of the algorithm especially at low signal-to-noise ratio (SNR) so as to be minimized or eliminated [9,10]. The technique of time reversal (TR), on the contrary, treats multipath a positive, which can self-adaptively modify the signal distortion caused by multipath effect and make the target get spatial-temporal focusing at source location [11], the more the better [12]. For the concrete theory and methods on TR readers can refer to the reviews of [13,14,15]. The self-focusing nature of TR has subsequently been verified in ultrasonic [16], acoustic [17], underwater acoustic [18,19,20] and electromagnetic fields [21,22] and achieved extensive applications in imaging [23], detection [24,25,26], localization [27,28], communication [29,30,31], DOA estimation [32] and other domains [33]. In this paper, we will focus on the DOA estimation by TR on multipath and low SNR conditions, and attempt to establish the TR multipath DOA model for underwater acoustics from the array signal processing perspective.

Asif’s research team has published many articles on this subject by active time reversal (ATR) in radar domain [32,34,35,36,37,38,39]. In [32] and [34], a TR DOA estimator for a passive target in a Ground Penetrating Radar (GPR) and a mobile communication network are studied, respectively. The performance of the DOA estimator with and without TR is compared and the simulation results show that the performance of the DOA estimator with TR is superior to the conventional approach. Furthermore, TR/range estimator is added and the active array source location for a single target is accomplished [35]. In [36], a TR-based DOA estimation framework for multiple-input multiple-output (MIMO) radars is presented. Reference [37] introduces TR into the Angle-Doppler estimation in MIMO radars and the closed-form expressions of Cramér-Rao bound (CRB) for DOA and Doppler frequency of a moving target in MIMO and TR/MIMO are derived in [38]. Reference [39] applies the compressive sensing (CS) and TR to MIMO radars to achieve the joint estimation of DOA, direction of departure (DOD) and Doppler information. In addition, Fu [40] proposes a virtual time reversal (VTR) method for the DOA estimation of electromagnetic signal emitted from a single communication station passively, which can determine the azimuth of the radiation source by finding the maximum energy points in the scanning area. Ciuonzo et al. [41,42,43] studies the performance of multiple signal classification (MUSIC) for computational time reversal (computational TR is VTR [44,45]) applications. Reference [46] addresses a DOA estimation method by passive time reversal (PTR) in a low angle target parameter estimation and antenna array scenario. Song referred to VTR proposed in [47] as PTR in the overview of underwater TR communication [31] and we will follow this statement in the later discussion. More information on ATR, PTR and VTR will been discussed in Section 2. The above TR DOA estimation method in the electromagnetic field is useful for DOA estimation of underwater target by TR.

In the underwater scenario, Reference [48] utilizes PTR to study the DOA estimation performance of a uniform shallow sea target, which proposes a super-directional model based on non-uniform linear array (NLA), establishes the simulation model from the signal detection point of view and uses the conventional beamforming method to achieve the azimuth estimation at low SNR. A PTR target orientation algorithm under Doppler spread is proposed for underwater acoustic multipath time-varying channel in [49] and the target orientation can be realized accurately by frequency compensation and time reversal processing.

From the above discussions, we can see that the current TR DOA estimation methods mainly focus on the ATR and VTR, which have the following problems. First, the methods based on ATR needs two signal transmissions so that the attenuation of energy is greater and receiving SNR of the array is reduced, thus affecting the estimation performance, especially for the weak target. Then although there is only one receiving process in PTR, it needs to first solve the problem how to obtain the real channel exactly in that only when the analog channel and actual one are completely matched can the ideal TR spatial-temporal focusing effect and accurate DOA estimation be achieved. To solve the above problems, an improved method called virtual TR based on active detection (ADVTR) is proposed in [50] to accomplish uniform linear array (ULA) beamforming, which establishes the required model from the perspective of signal detection, and estimates the angle by conventional beamforming method. Combining the advantages of ATR with PTR, the first process of ADVTR will realize the detection and channel estimation in the real channel and the second process will complete TR focusing virtually in the computer. Compared with ATR, there is no real re-emitting process so that the second receiving process has no noise in ADVTR and the SNR of the array received signal are higher; Compared with PTR, the characteristics of ocean channels can been estimated ideally according to the active detection while avoiding the PTR approximation process on the basis of the same processing gain with PTR.

There are few references on the ADVTR method: in addition to [50], the method is also intended for target detection in [51]. Based on [50,51], we propose in this paper a DOA estimation method based on ADVTR for underwater multiple targets from array signal processing perspective. It can exploit the underwater multipath propagation channel to improve the SNR of the received signal and the accuracy of target DOA estimation, especially at low SNR. We believe the method proposed in this paper can be applied in underwater search & rescue, wreck salvage and other fields as well. The main contribution in this context is as follows:

(1) An ADVTR Capon method is proposed to improve the performance of DOA estimation at low SNR;
(2) The model of conventional multipath and ADVTR multipath for ULA are established based on underwater acoustics propagation theory and array signal processing theory;
(3) In contrast with the method in [50] which is only confirmed through simulation, the performance of ADVTR Capon algorithm is verified and analyzed by simulation and tank experiment;
(4) Our model and method in this paper are readily extended to the DOA estimation of two or more targets with respect to [50], while [50] can only achieve one target’s orientation.

The rest of this paper is organized as follows: the ADVTR method is introduced in Section 2. A system model based on multipath is deduced in details in Section 3. Section 4 reviews the spatial smoothing and conventional Capon algorithm firstly, and then presents the ADVTR Capon algorithm based on spatial smoothing. The performance of the proposed ADVTR Capon algorithm is analyzed using the simulated data from Bellhop acoustic field simulation in Section 5. Section 6 illustrates the anechoic tank experiment and the DOA estimation results. Conclusions and future works are given in Section 7.

Notation: Lower and upper case boldface letters denote vectors and matrices, respectively; ${\left(\cdot \right)}^{*}$, ${\left(\cdot \right)}^{T}$, ${\left(\cdot \right)}^{H}$ and ${\left(\cdot \right)}^{-1}$ stand for conjugate, transpose, Hermitian transpose and inverse, respectively; E{⋅} is the expected value of a random quantity; ⊗ is the convolution of two signal and ⊙ is the Hadamard product of two vectors or matrices, i.e., the vector or matrix of their components wise product.

## 2. The Principle of ADVTR

In this section, we mainly discuss the principle of ADVTR method and then give the flow diagram of the DOA estimation method based on ADVTR.

TR, known as phase conjugation (PC) in optics, is a process of transmitting the received signal on an array in a time-reversed order, and then due to spatial reciprocity and TR invariance of the linear acoustic wave equation, the retransmitted signal converges back to the position where the original signal was generated [31]. If the retransmitted signal is actually transmitted over the real medium, it is referred to as a so-called physical TR, while if the back-propagation is computed or done numerically, in a fictitious, reference medium, it is called computational or virtual TR [43,44]. Dowling [52] first proposed the concept of ATR and PTR and pointed out that PTR processing is related to ATR by invoking reciprocity for a measured acoustic signal as a replacement for the back-propagation step of ATR. Therefore, according to whether the process of back-propagation is performed in the real medium, PTR is also regarded as VTR.

**Figure 1:**
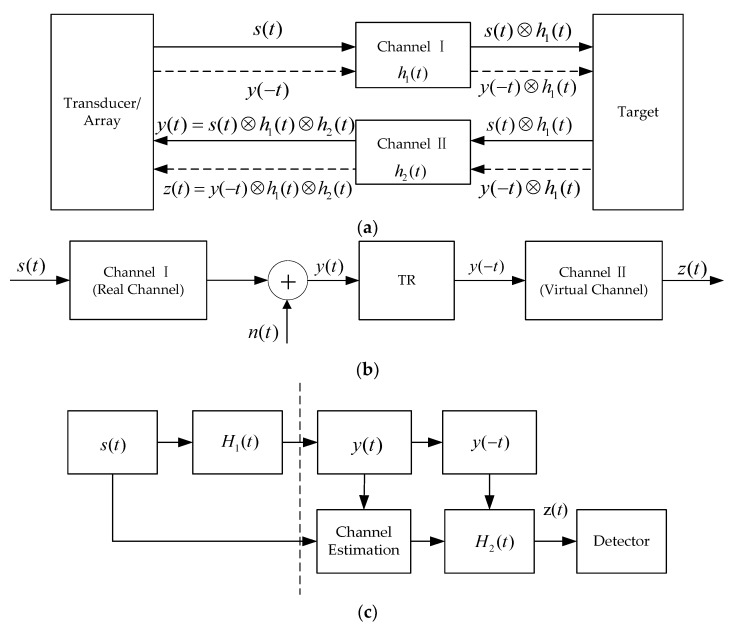
Diagram of ATR, PTR and ADVTR. (**a**) ATR; (**b**) PTR; (**c**) ADVTR.

ATR, PTR and ADVTR have been used for target detection in [51,53,54]. Here we will refer to above publications to compare the difference between ATR, PTR and ADVTR. The diagrams of ATR, PTR and ADVTR are shown in Figure 1.

For ATR [53], as shown in Figure 1a, the process can be divided into two phases. During phase 1, the transducer emits a detection signal s(t) through the underwater acoustic channel $h_{1}(t)$ to illuminate possible targets and receives reflected signal y(t) through the channel $h_{2}(t)$. During phase 2, the time reversed signal y(−t) is re-transmitted to the same channel as phase 1 once again. The transducer receives the signal z(t) during phase 2.

For PTR [54], as shown in Figure 1b, the radiation signal from the target s(t) is received by the hydrophone through channel I, y(t) corresponds to the reception signal with the additive noise n(t), which is different with ATR in that it doesn’t send signal again and channel II is simulated by computer. Only when channel I and channel II match with each other, the energy focus can be achieved.

For ADVTR [51], as shown in Figure 1c, the dashed line of ATR in Figure 1a or phase 2 of ATR is done virtually in a computer. To illustrate the “active detection (AD)” and “virtual TR (VTR) ” of the method more clearly, the diagram is expressed as shown in Figure 1c, which is separated into two parts by the dash line. Firstly, in the active part, the transducer emits a detection signal s(t) through underwater acoustic channel $H_{1}(t)$ to the interested area and receives the reflected signal y(t) from a target. Then the virtual underwater acoustic channel $H_{2}(t)$ can be estimated by comparing the emitted signal s(t) with the received signal y(t) in the virtual part. Finally, the time reversed signal y(−t) is transmitted virtually through the virtual channel $H_{2}(t)$ and detected by the detector. Note, $H_{1}(t)$ in (c) is equal to $h_{1}(t)⊗h_{2}(t)$ in (a).

The principles on ATR and PTR can refer to [53,54]. Here we only discuss the ADVTR.

The received signal y(t) can be expressed as:

(1) $$y(t)=s(t)⊗H_{1}(t)+n(t)$$

where n(t) denotes environment noise.

Virtual time reversal is carried out for (1), and then the signal z(t) can be expressed as:

(2) $$z(t)=s(-t)⊗H_{1}(-t)⊗H_{2}(t)+n(-t)⊗H_{2}(t)$$

If the virtual channel $H_{2}(t)$ could match the true channel $H_{1}(t)$, i.e., $H_{1}(t)=H_{2}(t)=H(t)$, Equation (2) can be rewritten as:

(3) $$z(t)=s(-t)⊗\int_{-\infty }^{+\infty }h^{2}(t)dt+n(-t)⊗h_{2}(t)$$

From Equation (3), we can observe that time reversal could utilize the multi-path effect to strengthen the detected signal, and consequently improve the detection performance. The other observation from Equation (3) is that the output of ADVTR process has the same expression as PTR. Obviously, ADVTR reduces the number of transmissions to one, and has more focusing ability compared with ATR.

**Figure 2:**
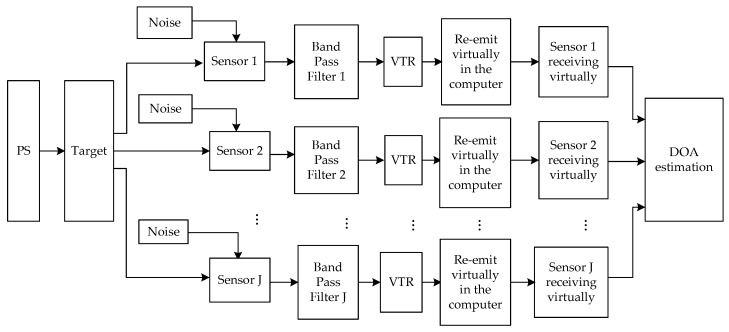
Flow diagram of the DOA estimation based on ADVTR.

Furthermore, combined with DOA estimation, we can obtain the flow diagram of the DOA estimation based on ADVTR as shown in Figure 2. First, PS transmits a detection signal to illuminate possible targets actively; then the sensors receive reflected signald and the environment noise should be considered. Several operations will be performed in the computer virtually, including filtering, channel estimating, VTR, re-emitting, receiving secondly and DOA estimation. Because of the AD, the frequency of received signal has been known, then filtering is performed before VTR and the virtual underwater acoustic channel can be estimated by comparing the emitted signal with the received signal. The TR signal of each sensor is re-emitted to the estimated channel virtually in the computer and the sensors receive the reflected signal once again and DOA estimation is done finally.

## 3. Multipath DOA Estimation Model for ULA Based on ADVTR

Based on underwater acoustics propagation theory and array signal processing theory, we derive the conventional multipath and ADVTR multipath DOA model for ULA respectively in this section.

### 3.1. Multipath Model Diagram for ULA

**Figure 3:**
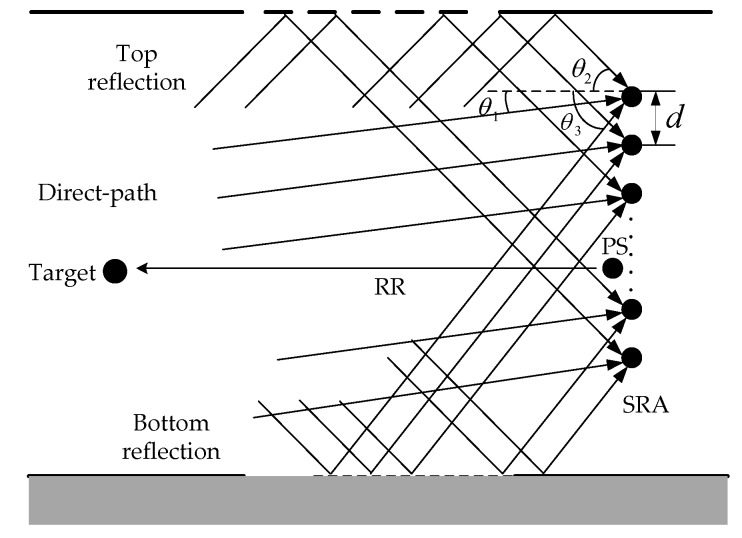
Multipath DOA estimation diagram for ULA.

The multipath DOA estimation model diagram for ULA based on the ray theory is shown in Figure 3. SRA in Figure 3 is the vertical ULA, whose element number is J and interelement spacing is d. For convenience, only three propagation paths between the target and SRA are drawn: the direct-path, the top-reflection-path and the bottom-reflection-path, whose incident angle (i.e., DOA of backscatter from target propagating via various paths) are as follows: $\theta_{1}$, $\theta_{2}$ and $\theta_{3}$. To be more general, the acoustic propagation paths deduced subsequently are not confined to these three paths, but N or M.

### 3.2. Conventional Multipath DOA Model for ULA

Firstly, PS located in element k of SRA will transmit the signal f(t). According to ray theory, the channel transfer function between the transmitting acoustic source and the target can be expressed as:

(4) $$h_{k}(t)=\sum_{n=1}^{N}c_{kn}\delta (t-\tau_{kn})$$

where N is the total number of sound ray; $c_{kn}$ is the amplitudes (or attenuation) of n-th eigen ray (also regarded as the propagation path) from element k to target; and $\tau_{kn}$ is the travel times (or delay) of n-th eigen ray from element k to target. On the basis of (4), the signal received by the target can be expressed as (5):

(5) $$x_{k}(t)=f(t)⊗h_{k}(t)=\sum_{n=1}^{N}c_{kn}f(t-\tau_{kn})$$

Assuming that the target reflection coefficient is 1, and considering the effect of noise from reception process, the signal received by the element j is:

(6) $$\begin{array}{ll} y_{j}(t) & =x_{k}(t)⊗h_{j}(t)+v_{j}(t)\\ & =\sum_{n=1}^{N}c_{kn}f(t-\tau_{kn})⊗\sum_{m=1}^{M}c_{jm}\delta (t-\tau_{jm})+v_{j}(t)\\ & =\sum_{m=1}^{M}\sum_{n=1}^{N}c_{jm}c_{kn}f(t-\tau_{jm}-\tau_{kn})+v_{j}(t) \end{array}$$

where $h_{j}(t)$ represents the channel function between the target and element j; $v_{j}(t)$ is the environment noise received by element j. $c_{jm}$ and $\tau_{jm}$ represent amplitudes and travel times of the m-th propagation path from target to element j separately.

Suppose the transmitter signal as $f(t)=s(t)e^{j\omega_{c}t}$. Combining far field and narrow band model theory of array signal processing [1], Equation (6) can be expressed as:

(7) $$\begin{array}{ll} y_{j}(t) & =\sum_{m=1}^{M}\sum_{n=1}^{N}c_{jm}c_{kn}f(t-\tau_{kn}-\tau_{1m}-\Delta \tau_{jm})+v_{j}(t)\\ & \approx \sum_{m=1}^{M}\sum_{n=1}^{N}c_{jm}c_{kn}s(t)e^{j\omega_{c}(t-\tau_{kn}-\tau_{1m}-\Delta \tau_{jm})}+v_{j}(t)\\ & =\sum_{m=1}^{M}c_{jm}e^{-j\omega_{c}\Delta \tau_{jm}}e^{-j\omega_{c}\tau_{1m}}\cdot \sum_{n=1}^{N}c_{kn}e^{-j\omega_{c}\tau_{kn}}\cdot s(t)e^{j\omega_{c}t}+v_{j}(t) \end{array}$$

where $\tau_{1m}$ represents the reference delay associated with backscatter from target traveling via path m to element 1 (the reference element); and $\Delta \tau_{1m}$ is the interelement delay associated with path m originating from target in excess of $\tau_{1m}$ with respect to the receiving element j.

The received signals of element j expressed in (7) is generalized to other array elements and expressed in the matrix form as:

(8) $$Y(t)=(C⊙A)DX_{k}F(t)+V(t)$$

where the symbol “⊙” is the Hadamard Product or Basic Product, which can realize the point-to-point product of two identical-order matrices; $Y(t)={\left[y_{1}(t),\cdots ,y_{J}(t)\right]}^{T}$ is defined as the received signal matrix received by the element 1−J of the SRA; C is a (J×M) matrix representing the attenuation matrix with respect to the channel response function between the target and SRA elements, whose element $c_{jm}$ indicates the attenuation associated with backscatter from target traveling via path m to element j; A is a (J×M) matrix representing a relative delay matrix to the reference element (element 1) and given by (9); $D={\left[e^{-j\omega_{c}\tau_{11}},e^{-j\omega_{c}\tau_{12}},\cdots ,e^{-j\omega_{c}\tau_{1M}}\right]}^{T}$ is the reference delay matrix including interrelated delay from target traveling via all M paths to element 1; $X_{k}$ expressed as Equation (10) can be considered as a target-received signal matrix which is transmitted from the element j through the channel to the target; $F(t)=s(t)e^{j\omega_{c}t}$ is called the transmitting matrix; and V(t) indicates the noise matrix:

(9) $$A=\left[\begin{array}{cccc} e^{-j\omega_{c}\Delta \tau_{11}} & e^{-j\omega_{c}\Delta \tau_{12}} & \cdots & e^{-j\omega_{c}\Delta \tau_{1M}}\\ e^{-j\omega_{c}\Delta \tau_{21}} & e^{-j\omega_{c}\Delta \tau_{22}} & \cdots & e^{-j\omega_{c}\Delta \tau_{2M}}\\ \vdots & \vdots & \vdots & \vdots \\ e^{-j\omega_{c}\Delta \tau_{J1}} & e^{-j\omega_{c}\Delta \tau_{J2}} & \cdots & e^{-j\omega_{c}\Delta \tau_{JM}} \end{array}\right]$$

(10) $$X_{k}=\sum_{n=1}^{N}c_{kn}e^{-j\omega_{c}\tau_{kn}}=(C(k,:)⊙A(k,:))D$$

Referring to array signal processing theory [1], when the array interelement spacing of ULA is d, Equation (9) can be represented as (11):

(11) $$\begin{array}{ll} A & =[a(\theta_{1}),a(\theta_{2}),\cdots ,a(\theta_{M})]\\ & =\left[\begin{array}{cccc} 1 & 1 & \cdots & 1\\ e^{-j\frac{2\pi }{\lambda }d\sin\theta_{1}} & e^{-j\frac{2\pi }{\lambda }d\sin\theta_{2}} & \cdots & e^{-j\frac{2\pi }{\lambda }d\sin\theta_{M}}\\ \vdots & \vdots & \vdots & \vdots \\ e^{-j\frac{2\pi }{\lambda }(J-1)d\sin\theta_{1}} & e^{-j\frac{2\pi }{\lambda }(J-1)d\sin\theta_{2}} & \cdots & e^{-j\frac{2\pi }{\lambda }(J-1)d\sin\theta_{M}} \end{array}\right] \end{array}$$

A of Equation (9) is known as the array steering matrix (DOA matrix) that contains all multipath information, and mainly depends on the array structure and the direction of arrival, where m-th column $a(\theta_{m})$ is known as steering vector and denotes the received information from target via path m to each element; $\theta_{m}$ is the angle information of path m from target to each element.

As mentioned, the method proposed in the paper is to add some additional stages such as TR, re-emitting, reflecting by target and receiving of SRA on the basis of conventional DOA estimation. Therefore the following discussion will devote to the establishment of a multipath DOA model based on ADVTR.

### 3.3. ADVTR Multipath DOA Model for ULA

According to the flow of ADVTR mentioned in Section 2, the first process of ADVTR is active detection, so the frequency of the transmitted signal has been known when the array receives the signal. Then the filter can be introduced to eliminate the impact of noise before TR operation. Subsequently VTR is performed virtually for the received signals of each SRA in the computer, and taking element j as an example:

(12) $$y_{j}(-t)=B\cdot s(-t)e^{-j\omega_{c}t}=Bf(-t)$$

where B is expressed as (13), whose value is independent of time t and can be considered as a coefficient:

(13) $$B=(\sum_{m=1}^{M}c_{jm}e^{-j\omega_{c}\Delta \tau_{jm}}e^{-j\omega_{c}\tau_{1m}})\cdot \sum_{n=1}^{N}c_{kn}e^{-j\omega_{c}\tau_{kn}}$$

The signal expressed as (12) is energy normalized and then re-transmitted virtually into the channel as a secondary transmission signal in the computer, which still satisfies the far field and narrow band model theory. Repeat the above first procedure, refer to Equations (4)–(7), and suppose the signal received by element l is $z_{l}(t)$:

(14) $$\begin{array}{ll} z_{l}(t) & \approx \sum_{m=1}^{M}\sum_{n=1}^{N}c_{lm}c_{jn}g_{j}Bs(-t)e^{-j\omega_{c}(t-\tau_{jn}-\tau_{1m}-\Delta \tau_{lm})}\\ & =\sum_{m=1}^{M}c_{lm}e^{j\omega_{c}\Delta \tau_{lm}}e^{j\omega_{c}\tau_{1m}}\cdot \sum_{n=1}^{N}c_{jn}e^{j\omega_{c}\tau_{jn}}\cdot g_{j}Bs(-t)e^{-j\omega_{c}t} \end{array}$$

where $g_{j}$ is the normalized coefficient of element j obtained with reference to the transmitted signal power, and owing to the principle of virtual time reversal, the secondary process has no noise.

Referring to (8)–(11), the received signal of all of elements of SRA on the second time with element j probing virtually is:

(15) $$Z_{j}(t)=(C⊙A^{*})D^{*}X_{j}{}^{*}g_{j}y_{j}(-t)$$

where A, C, D are the same as the parameters of (5), and $X_{j}$ is the same as $X_{k}$ of (8) except that $X_{k}$ is the received signal matrix with element k probing, $X_{j}$ is the received signal matrix with element j probing.

The received signals with element j of SRA probing expressed as (15) are generalized to other array elements and the sum of received signals of SRA after TR is:

(16) $$\begin{array}{ll} Z_{\mathrm{tr}} & =\sum_{j=1}^{J}Z_{j}(t)\\ & =\sum_{j=1}^{J}\left(C⊙A^{*})D^{*}X_{j}{}^{*}g_{j}y_{j}(-t\right)\\ & =(C⊙A^{*})D^{*}\sum_{j=1}^{J}X_{j}{}^{*}g_{j}y_{j}(-t) \end{array}$$

## 4. DOA Estimation Algorithm

In order to express our method clearly, we briefly review the spatial smoothing and conventional Capon algorithm. Then, we give our DOA estimation algorithm in detail and the computational complexity of the proposed algorithm.

### 4.1. Spatial Smoothing Algorithm

Since the multipath model is introduced, the signals arriving to SRA from the target via each path are coherent signals. Therefore, spatial smoothing technique is applied into the coherent processing prior to the DOA estimation [55].

**Figure 4:**
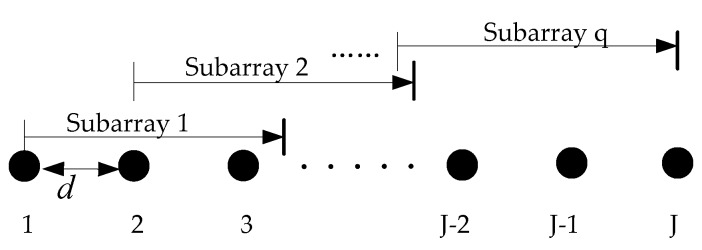
Spatial smoothing algorithm diagram.

In combination with the above model in Section 3, suppose that the array is an equidistant linear array with J elements and element distance d. The J elements linear array is decomposed into q overlapping subarrays, and each subarray has ns array elements, as shown in Figure 4. In Figure 4, the relationship with J, ns and q should satisfy the relationship of Equation (17):

(17) q=J+1−ns

The subarray i is composed of line i to line i+ns−1 of the signal matrix, and taking the array received signal Y in (8) as an example, the signal matrix of subarray i can be represented as:

(18) $$Y_{i}(t)={\left[y_{i}(t),y_{i+1}(t),\cdots ,y_{i+ns-1}(t)\right]}^{T}$$

The covariance matrix can be calculated for each subarray of (18) by:

(19) $$R_{Y_{i}}=E\{Y_{i}(t)Y_{i}{\left(t\right)}^{H}\}$$

Then, the spatial smoothing covariance matrix of the received signal for the first time can be obtained by averaging (19) which is expressed as (20):

(20) $$R_{YY}=\frac{1}{q}\sum_{i=1}^{q}R_{Y_{i}}$$

Similarly, the signals received secondly shown in (16) are smoothed spatially, and the signal matrix of subarray i can be represented as:

(21) $$Z_{tri}(t)={\left[Z_{tr}(i),Z_{tr}(i+1),\cdots ,Z_{tr}(i+ns-1)\right]}^{T}$$

The covariance matrix can be calculated for each subarray of (21) by:

(22) $$R_{Z_{i}}=E\{Z_{\mathrm{tri}}(t)Z_{\mathrm{tri}}{\left(t\right)}^{H}\}$$

The spatial smoothing covariance matrix of the signal received secondly can be expressed as (23) by averaging (22):

(23) $$R_{ZZ}=\frac{1}{q}\sum_{i=1}^{q}R_{Z_{i}}$$

### 4.2. Conventional Multipath Capon Algorithm

According to the array signal processing theory [1], the purpose of the Capon algorithm is attempting to minimize the power by noise and other interference signal except the desired signal, while maintaining a fixed gain in the direction from which the signal originated. Combining the model established by Equation (8), the Capon algorithm can be represented as the following minimum problem:

(24) $$\begin{array}{l} \min w^{H}(\theta ){\hat{R}}_{Y}w(\theta )\\ S.T.:\;w^{H}(\theta )a(\theta_{1})=1 \end{array}$$

where ${\hat{R}}_{Y}=\frac{1}{N}E\{Y(t)Y{\left(t\right)}^{H}\}$ is the sample covariance matrix of the array output signal with the multipath; $\theta_{1}$ is desired angle information and w is the weight value.

The Lagrange multiplier method can be used to obtain the solution of the above problem:

(25) $$w=\frac{{\hat{R}}_{Y}{}^{-1}a(\theta_{1})}{a^{H}(\theta_{1}){\hat{R}}_{Y}{}^{-1}a(\theta_{1})}$$

Equation (26) can be obtained by taking Equation (25) into Equation (24), which means the largest power in the desired direction while the power except the desired direction is the smallest:

(26) $$P(\theta )=\frac{1}{a^{H}(\theta_{1}){\hat{R}}_{Y}{}^{-1}a(\theta_{1})}$$

Thus the spatial spectrum of the Capon algorithm can be defined as:

(27) $$P(\theta )=\frac{1}{a^{H}(\theta ){\hat{R}}_{Y}{}^{-1}a(\theta )}$$

where a(θ) is the steering vector and can be defined as (28):

(28) $$a(\theta )={\left[1,e^{-j\frac{2\pi }{\lambda }d\sin\theta },\cdots ,e^{-j\frac{2\pi }{\lambda }(J-1)d\sin\theta }\right]}^{T}$$

### 4.3. ADVTR Capon Algorithm

According to Equation (16), follow the same steps as the above conventional DOA estimation:

(29) $$\begin{array}{l} \min w_{tr}{}^{H}(\theta ){\hat{R}}_{Z_{tr}}w_{tr}(\theta )\\ \mathrm{subject}\;\mathrm{to}:\;w_{tr}{}^{H}(\theta )a_{tr}(\theta_{1})=1 \end{array}$$

Equation (29) is similar to (24) but ${\hat{R}}_{Z_{tr}}=\frac{1}{N}E\{Z_{tr}(t)Z_{tr}{\left(t\right)}^{H}\}$ is the sample covariance matrix after virtual time reversal output signal and the other values are the same as above. At this time, the spatial spectrum of the ADVTR Capon algorithm is:

(30) $$P_{tr}(\theta )=\frac{1}{a_{{}^{tr}}^{H}(\theta ){\hat{R}}_{Z_{tr}}{}^{-1}a_{tr}(\theta )}$$

where $a_{tr}(\theta )$ in (30) can be defined as (31):

(31) $$a_{tr}(\theta )={\left[1,e^{j\frac{2\pi }{\lambda }d\sin\theta },\cdots ,e^{j\frac{2\pi }{\lambda }(J-1)d\sin\theta }\right]}^{T}$$

### 4.4. Spatial Smoothing Capon and ADVTR Capon Algorithm

The spatial smoothing technique is applied to the Capon algorithm and ADVTR Capon algorithm respectively. For the Capon algorithm: ${\hat{R}}_{Y}$ in (27) is replaced by $R_{YY}$ in (20) and a(θ) in (27) is replaced by the steering vector of each subarray (i.e., the dimensions of a(θ) are the same as those of the subarray) when spectral peak is being searched. So the spectrum function for spatial smoothing Capon algorithm can be obtained:

(32) $$P_{s}(\theta )=\frac{1}{a_{s}^{H}(\theta )R_{YY}{}^{-1}a_{s}(\theta )}$$

The DOA value of the target can be gained by searching the spectral peak of the Equation (32), in which $a_{s}(\theta )$ is the steering vector for spatial smoothing Capon algorithm expressed as (33):

(33) $$a_{s}(\theta )={\left[1,e^{-j\frac{2\pi }{\lambda }d\sin\theta },\cdots ,e^{-j\frac{2\pi }{\lambda }(ns-1)d\sin\theta }\right]}^{T}$$

For the ADVTR Capon algorithm, ${\hat{R}}_{Z_{tr}}$ and $a_{tr}(\theta )$ in (30) are replaced by $R_{ZZ}$ in (23) and the steering vector $a_{trs}(\theta )$ of each subarray when spectral peak is being searched. So the spectrum function for spatial smoothing ADVTR Capon algorithm can be obtained:

(34) $$P_{trs}(\theta )=\frac{1}{a_{trs}^{H}(\theta )R_{ZZ}{}^{-1}a_{trs}(\theta )}$$

The DOA value of the target can be gained by searching the spectral peak of the (34), in which $a_{trs}(\theta )$ is the steering vector for spatial smoothing ADVTR Capon algorithm expressed as (35):

(35) $$a_{trs}(\theta )={\left[1,e^{j\frac{2\pi }{\lambda }d\sin\theta },\cdots ,e^{j\frac{2\pi }{\lambda }(ns-1)d\sin\theta }\right]}^{T}$$

### 4.5. Computational Complexity of Smoothing Capon and ADVTR Capon Algorithm

Assuming that the number of snapshot is L, the grid is divided into K angles when searching for spatial spectrum. Other parameters can be seen in Section 3 and Section 4. We mainly consider the operations on matrix and vector, ignoring other operations. The computational complexities of the conventional Capon and ADVTR Capon algorithm are shown in Table 1.

From the previous discussions, since the additional processes are introduced in ADVTR Capon algorithm, like VTR, re-emitting virtually and re-receiving virtually (see Figure 2), its computational complexity is higher than the conventional Capon algorithm. Considering the performance improvement provided by the ADVTR Capon algorithm, its computational complexity is acceptable.

## 5. Simulation Results

In this section we confirm our theoretical model through simulations. The simulation environment is as follows: shallow water waveguide, uniform sound speed environment (the sound speed is 1.5 km/s), the bottom is to be modeled as an Acousto-Elastic halfspace, the density is 2 kg/m^3^ and the case of single objective. The simulation model has been shown in Figure 3, where the number of TRM (SRA) is 9, the interelement spacing is 0.75 m, the element 1 is 75 m from the sea surface, the depth of bottom is 500 m, the source depth is 78 m and the range between PS and the target is 2 km. In order to verify the validity of the proposed algorithm, two angles such as 0° and −5° are selected randomly to perform the simulations apart. In other words, target is located in 78 m and 253 m respectively. The signal transmitted by PS is a 1 kHz continuous wave (CW) signal. The simulation environment is shown in Table 2.

Bellhop special acoustic field simulation toolbox under MATLAB simulation environment is used to simulate the ocean sound field environment. The multiple reflection path by sea surface or bottom are ignored because the energy attenuation is too large, and only the three paths illustrated in Figure 3 are considered, then the various parameters used for simulation are obtained, as shown in Table 3 and Table 4.

Through the simulation, we can discover that the amplitudes of the same paths from the target to each element (e.g., the amplitude from the target to each element via the top-reflection) are very closer. Then for convenience, the amplitude information between PS and the target expressed in the table is the corresponding direct-path, top-reflection-path and bottom-reflection-path successively.

In order to compare the performance of the algorithm with or without ADVTR at the same conditions, SNR is defined as the ratio of signal to noise when the signal is received at the first time; while the noise doesn’t added to the received signal on the second time on account of ADVTR.

**Figure 5:**
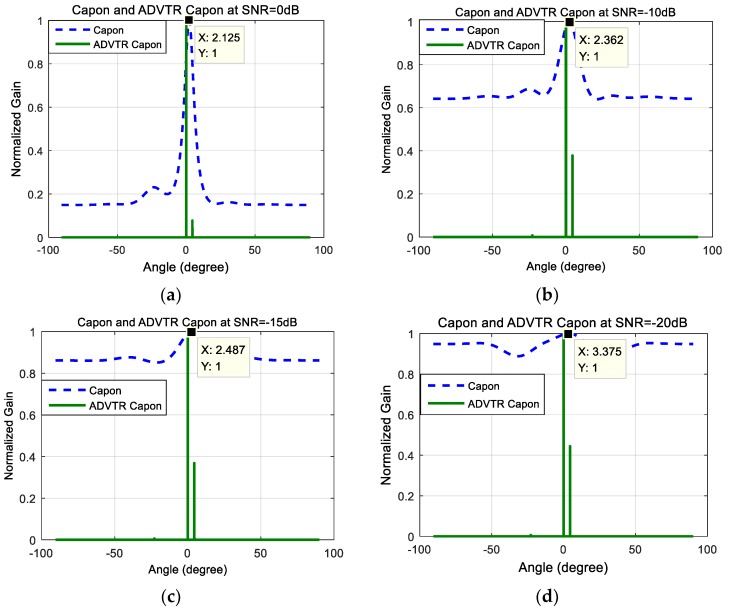
Capon estimator and ADVTR Capon estimator at $\theta =0^{{}^{\circ}}$. (**a**) SNR = 0 dB; (**b**) SNR = −10 dB; (**c**) SNR = −15 dB; (**d**) SNR = −20 dB.

**Figure 6:**
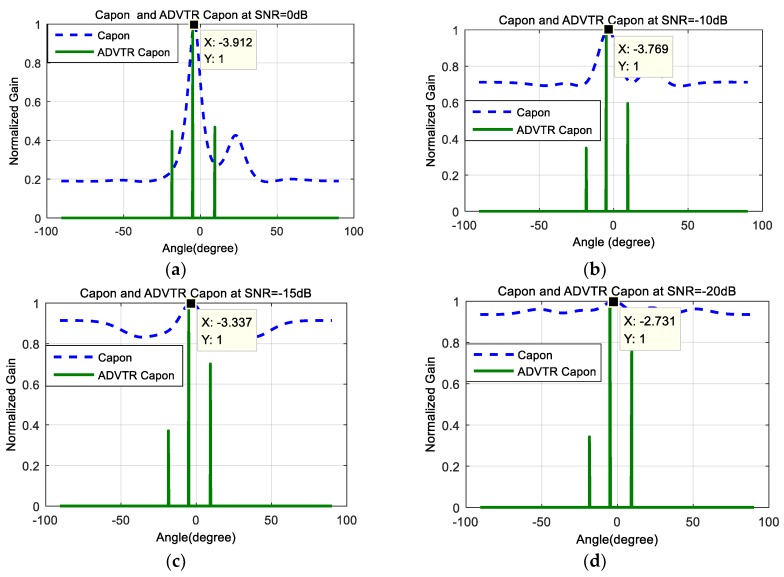
Capon estimator and ADVTR Capon estimator at $\theta =-5^{{}^{\circ}}$. (**a**) SNR = 0 dB; (**b**) SNR = −10 dB; (**c**) SNR = −15 dB; (**d**) SNR = −20 dB.

Figure 5a–d shows the DOA estimation performance comparison with Capon estimator and ADVTR Capon estimator for target at 0° when the SNR is 0 dB, −10 dB, −15 dB, and −20 dB; Figure 6a–d shows the DOA estimation performance comparison with Capon estimator and ADVTR Capon estimator for target at −5° when the SNR is 0 dB, −10 dB, −15 dB and −20 dB. It can be seen from Figure 5 and Figure 6 that:

(1) Relative to the Capon algorithm, the ADVTR Capon algorithm can estimate accurately the expected value of the target, whose energy of the main lobe is far higher than its corresponding sidelobes and resolution is higher whether the target is at 0° or −5°. Taking a SNR of −10 dB plotted in Figure 6b when target is at −5° as an example, we can find that ADVTR Capon estimator has three peaks which correspond to the order of the multipath, whereas the conventional Capon estimator has only one peak and other two pieces of multipath information are lost. In addition, the ADVTR Capon estimator is more accurate with the highest peak in its spectrum observed at −5°, much closer to the simulated DOA of 4.998° and with much finer resolution (smaller lobes). The conventional Capon DOA estimator has a value of −3.769°.
(2) The main reason for the result (1) is that relative to the conventional Capon algorithm, the operation of TR and re-transmitting to the channel in ADVTR is performed in the computer, it will be focused virtually on the target according to the focusing characteristics of TR. The process is equivalent to the beamforming process used in the array signal processing, but TR method can focus the beam towards the DOA taking advantage of multipath and its adaptive focusing characteristic, thus the energy focusing on the target is greater than beamforming. Therefore, the DOA estimation angle is more accurate when ADVTR is introduced.
(3) With the change of SNR from 0 dB to −20 dB, the relations between main lobe and sidelobes for both the Capon and ADVTR Capon algorithms are the same that the energy of the sidelobes is higher and higher, and is getting closer to the main lobe. The difference is that the resolution for Capon algorithm is getting lower and lower, and the estimation deviation is more and more greater. However, the resolution for ADVTR Capon is almost not affected, and the target angle can be estimated without bias.
(4) For the ADVTR method, the variation is mainly concentrated on the relation between the main lobe and sidelobes when SNR varies, and the main lobe energy can be accurately focused on the direct-path, so the estimated DOA values are almost unbiased. Because of above reasons the root mean square error (RMSE) is not analyzed. The main reason for this result is that the emphasis of this paper is the performance improvement of the Capon DOA estimation algorithm when the ADVTR method is introduced, rather than the channel estimation method, so the channel used in the focusing is the same as active detection one in the simulation and the channel estimation process is not carried out so that the effect of focusing is very ideal. If the channel estimation process is added before the virtual focusing is implemented in the virtual time reversal experiment, the focusing effect may go to the bad, and the result of the DOA estimation will be affected.

## 6. Experiment Results

**Figure 7:**
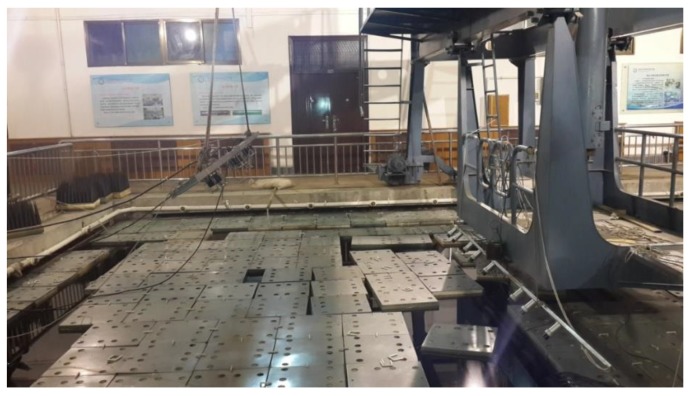
The anechoic tank laboratory.

**Figure 8:**
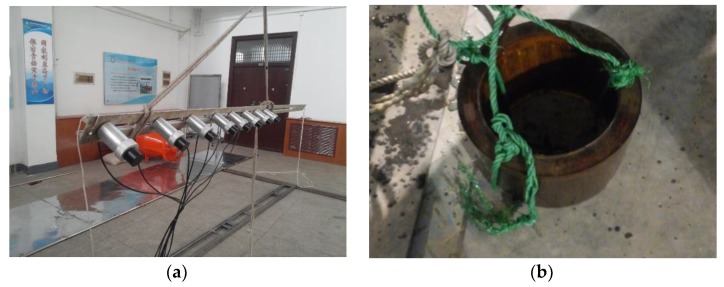
(**a**) The hydrophone array (**b**) The underwater acoustic transducer.

**Figure 9:**
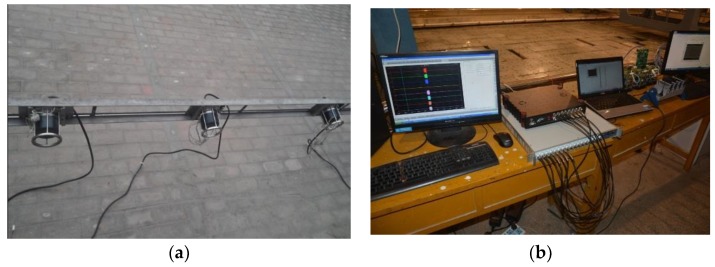
(**a**) The transceiver (**b**) The data acquisition field.

**Figure 10:**
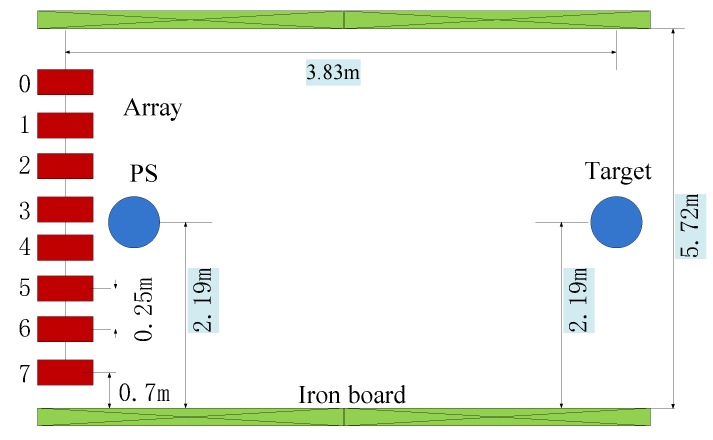
The data acquisition field.

To further verify the effectiveness of the proposed algorithm, a real set of data deriving from the experiment conducted in the anechoic tank laboratory in 2015 is employed for DOA estimation in this section. The experiment setup is shown in Figure 7. Figure 8 shows the hydrophone array and underwater acoustic transducer (PS). Figure 9 is the transceiver and data acquisition field. All the sensors in our experiment are made from piezoelectric ceramics. The experiment diagrammatic sketch is shown in Figure 10.

The test process is as follows: PS distributed in the center of the hydrophone array transmits a signal. In the real environment, the target is irradiated by the signal to produce the echo and then is received by the hydrophone array; while in the tank experiment, the transceiver is used to simulate the target echo: it will receive the transmitting signal from PS, scale it and return it into the tank to be received by the hydrophone array finally.

The whole device is placed horizontally in the tank and two pieces of iron sheet are adopted at the edge of the hydrophone array and transceiver to imitate the sea surface and floor respectively. The depth of both the array and transceiver is 0.8 m, and the range is 3.83 m. The PS and transceiver are aligned to the array center. The number of the array is 8, and the interelement spacing is 0.25 m. The PS transmitted a CW signal whose frequency is 3 kHz and width is one cycle. The overall experiment conditions are shown in Table 5.

**Figure 11:**
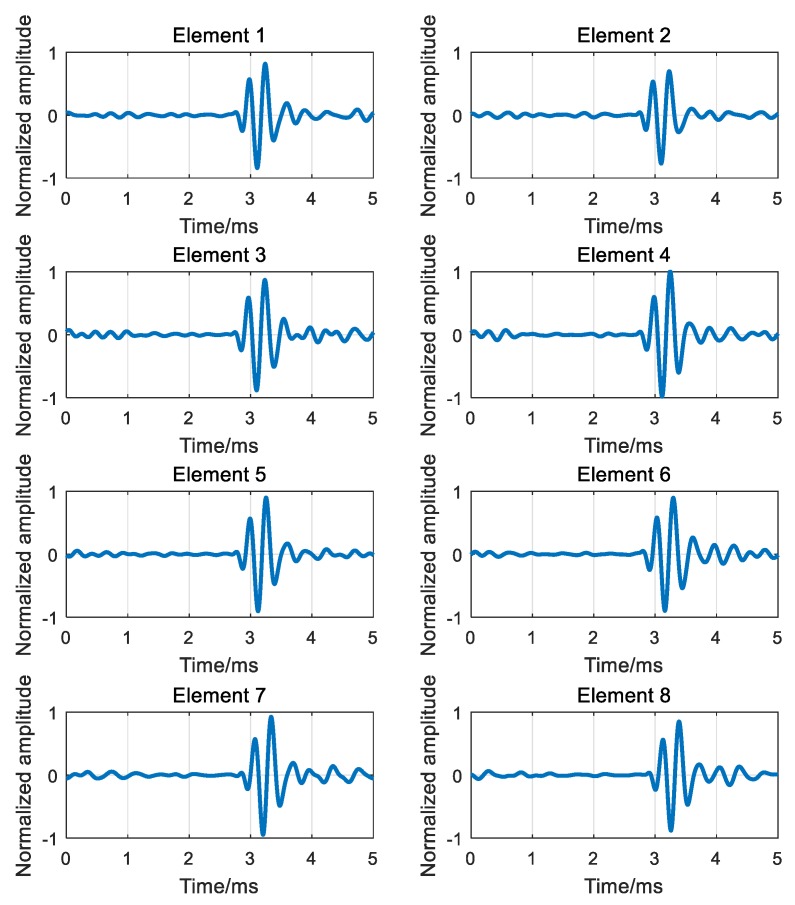
The first received signals of element 1–8.

The first received signals of each element are shown in Figure 11. It can be seen from Figure 11 that the due to the multipath effects of the channel, transmitting signal of one cycle has different degrees of delay expansion at each receiving element through the channel, and the receiving signals of each element in Figure 11 can be understood as the superposition of the signals corresponding to multiple paths.

**Figure 12:**
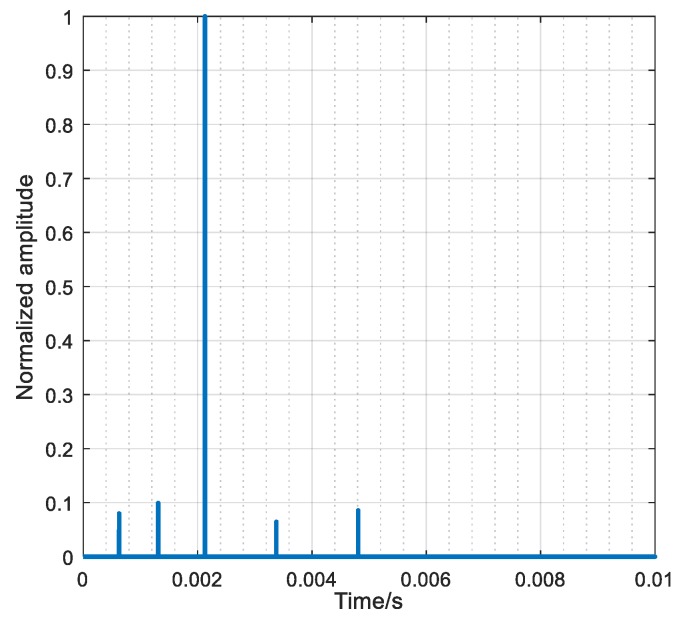
The estimated channel transfer functions between the target and element 2.

By calculating the correlation between the received signal shown in Figure 11 and PS, the channel response functions between the target and each element can be obtained, as illustrated in Figure 12, which only takes the element 2 as an example and the other elements are similar to it. According to the ray model, each line in Figure 12 represents a path; meanwhile the horizontal axis and the vertical axis represent the time and amplitude of the path respectively. As can be seen from Figure 12, there are 5 paths between the target and the element 2.

**Figure 13:**
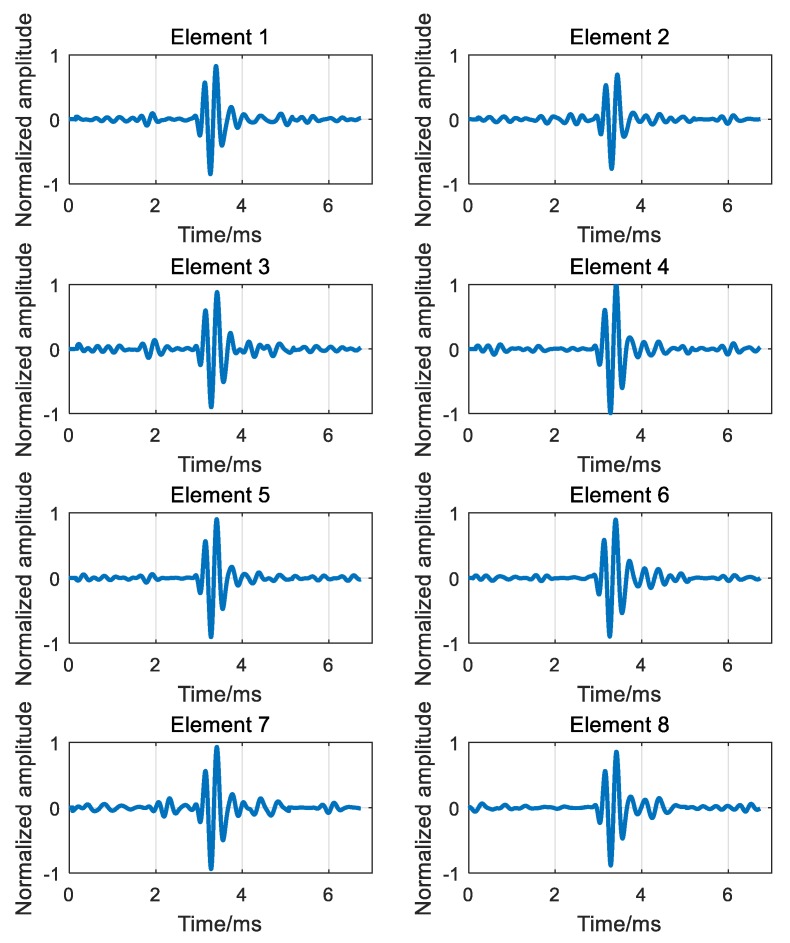
The second received signals of element 1–8.

According to the process of ADVTR, the first received signals of each element will be time reversed and then re-emitted virtually to the estimated channel; then the TR signals received secondly by each element can be obtained by convoluting the TR signals of Figure 11 and the estimated channel response functions illustrated in Figure 12 as shown in Figure 13.

By comparing the received signals before and after TR as shown in Figure 11 and Figure 13, it can be obtained that the received signals after TR are focused in time and the delay difference caused by multipath is overcome to a certain extent.

**Figure 14:**
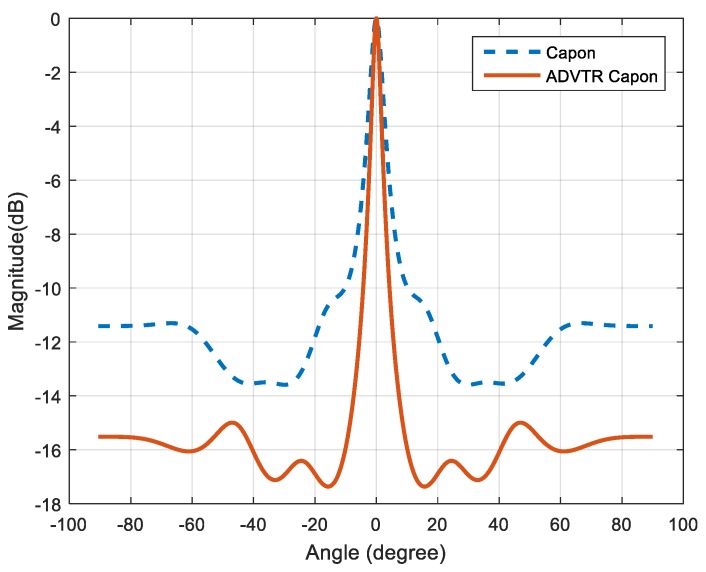
Capon estimator and ADVTR Capon estimator in the tank.

For the signals shown in Figure 11 and Figure 13, the covariance matrices re-calculated individually and the algorithms proposed in Section 4 are employed to estimate the DOA of the target. The estimation results are shown in Figure 14.

It can be seen from Figure 14 that both the two methods can estimate the target accurately, whose estimated values are −0.0020°. The reason for this result is that the experimental environment in the tank can be considered as a noiseless one, so there are basically no differences between the two methods. It’s only that the ADVTR method has a relatively lower sidelobes and higher resolution. This result verifies the effectiveness of ADVTR method to some extent.

**Figure 15:**
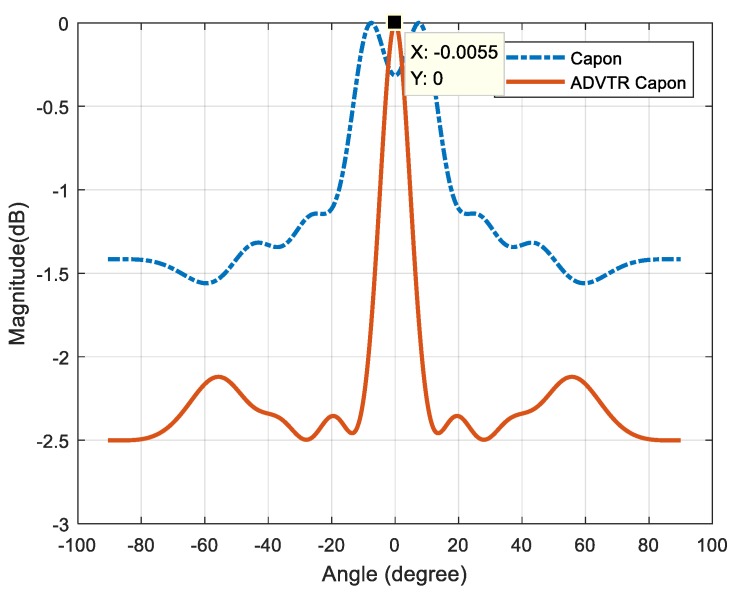
Capon estimator and ADVTR Capon estimator in the tank when SNR is −8 dB.

Next we will discuss the difference between the two methods under the noise environment which is much closer to the real situation. Therefore the Gauss white noise is artificially added to the first received signal. Then the above processes such as channel estimation, time reversal, the signals received secondly, the calculation of covariance matrix and DOA estimation are repeated. The performance comparison of both methods can be obtained as illustrated in Figure 15 when the SNR is −8 dB.

We can see from Figure 15 that when SNR is −8 dB the Capon estimator can’t estimate the angle of the target where two false peaks appear while the ADVTR Capon one is more accurate with the highest peak in its spectrum observed at −0.0055° which is much closer to the true value of 0° and with much finer resolution (smaller lobes).

**Figure 16:**
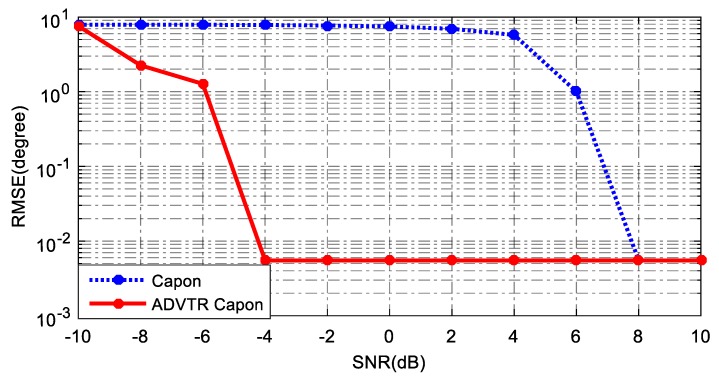
RMSE for Capon estimator and ADVTR Capon estimator in the tank.

Due to the randomness of noise, the estimation results of Figure 15 are not enough to illustrate the problem. Thus Monte Carlo simulation tests are introduced. Environmental noise with different intensity is added, and Monte Carlo simulation based on 1000 runs for each SNR with and without TR is performed, as shown in Figure 16.

As can be seen from Figure 16 that RMSE estimated by the ADVTR Capon algorithm is smaller than the conventional Capon algorithm, especially when the SNR is very low, the advantage of ADVTR Capon algorithm is particularly obvious. Figure 16 illustrates the good performance of the ADVTR DOA estimation model on low SNR.

## 7. Conclusions and Future Work

In this paper, we use the ADVTR method to study the performance of underwater target DOA in the presence of multipath, and a DOA estimation algorithm based on the ADVTR Capon algorithm is proposed. Firstly, based on the ray theory, a conventional multipath DOA estimation model and an ADVTR DOA estimation model for ULA are established, and then the model is estimated with the spatial smoothing Capon algorithm. The simulation results show that the DOA estimator including ADVTR, compared with the conventional method without TR, has a more accurately estimated value, a higher resolution and a stronger ability to suppress sidelobes, so that the target DOA estimation can be achieved in the case of low SNR and multipath. The tank experiment further verifies the effectiveness of the proposed method.

As part of the future work, we intend to extend the proposed approaches to the following points in the future:

(1) The DOA estimation in this paper is concentrated upon a single target, so the multi-objective estimation on TR will be carried out in the future research.
(2) The location of underwater target can be achieved combining range estimator on TR with DOA estimator in this paper, and the lake experimental research will be carried out in the future.
(3) The deduction in the paper is based on the narrow band signal, and the DOA estimation of broadband signal is the future research work.
(4) The Capon algorithm is applied for DOA estimation in this paper, and other popular algorithms such as MUSIC will be studied in the future work.

## Figures and Tables

**Table 1:** Computational complexity analysis.

Algorithm	Computational Complexity
Capon	$O(ns^{2}\times L)+O(ns^{3})+K\cdot O(ns^{2}+ns)$
ADVTR Capon	$J\cdot O\{J(M+L)\}+O(ns^{2}\times L)+O(ns^{3})+K\cdot O(ns^{2}+ns)$

**Table 2:** Simulation environment.

Simulation Condition	Parameter
Attenuation	2 (dB/m) kHz
Number of targets	1
Number of elements	9
Interelement spacing	0.75 m
The depth of element 1	75 m
The depth of bottom	500 m
The source depth	78 m
Target depth (0°)	78 m
Target depth (−5°)	253 m
The range	2 km
Number of multipath	3

**Table 3:** Simulation parameters target at 0°.

Simulation Parameter	Value
Direction of arrival	{4.458°, 0°, −22.884°}
Amplitude	{4.99 × 10^−4^, 5.0 × 10^−4^, 2.92 × 10^−4^}
Delay of element 1	{1.3372291, 1.3333348, 1.4479731} s

**Table 4:** Simulation parameters target at −5°.

Simulation Parameter	Value
Direction of arrival	{19.396°, −4.998°, −18.498°}
Amplitude	{4.93 × 10^−4^, 4.98 × 10^−4^, 4.74 × 10^−4^}
Delay of element 1	{1.351145, 1.3386036, 1.4065851} s

**Table 5:** Experiment environment.

Experiment Condition	Parameter
Distance between two pieces of iron	5.72 m
Number of elements	8
Interelement spacing	0.25 m
The position of elements 8 (from the bottom sheet)	0.7 m
Target position (from the bottom sheet)	2.19 m
PS position (from the bottom sheet)	2.19 m
Target depth	0.8 m
Array depth	0.8 m
The range	3.83 m
DOA	0°
PS frequency	3 KHz
Sampling frequency	1.04 MHz
